# Genetic Polymorphisms of P2RX7 but Not of ADORA2A Are Associated with the Severity of SARS-CoV-2 Infection

**DOI:** 10.3390/ijms25116135

**Published:** 2024-06-02

**Authors:** Jorge Lindo, Célia Nogueira, Rui Soares, Nuno Cunha, Maria Rosário Almeida, Lisa Rodrigues, Patrícia Coelho, Francisco Rodrigues, Rodrigo A. Cunha, Teresa Gonçalves

**Affiliations:** 1 FMUC—Faculty of Medicine, University Coimbra, 3004-504 Coimbra, Portugal; lindojorgester@gmail.com (J.L.); cnogueira@fmed.uc.pt (C.N.); rui.soares@uc.pt (R.S.); mralmeida2008@gmail.com (M.R.A.); 2 CNC-UC—Center for Neuroscience and Cell Biology, University Coimbra, 3004-504 Coimbra, Portugal; lisa1cor@gmail.com; 3 CIBB—Centre for Innovative Biomedicine and Biotechnology, University Coimbra, 3004-504 Coimbra, Portugal; 4 Clinical Pathology Service, Portuguese Oncology Institute of Coimbra, 3004-011 Coimbra, Portugal; nunocunha@ipocoimbra.min-saude.pt; 5 IPCB/ESALD—Instituto Politécnico de Castelo Branco, Escola Superior de Saúde Dr. Lopes Dias, SPRINT-IPCB—Sport Physical Activity and Health Research & Innovation Center, 6000-767 Castelo Branco, Portugal; patriciacoelho@ipcb.pt (P.C.); franciscobrodrigues@ipcb.pt (F.R.)

**Keywords:** adenosine A2A receptor, ATP P2X7 receptor, COVID-19, SARS-CoV-2, polymorphisms

## Abstract

SARS-CoV-2 infection ranges from mild to severe presentations, according to the intensity of the aberrant inflammatory response. Purinergic receptors dually control the inflammatory response: while adenosine A2A receptors (A2ARs) are anti-inflammatory, ATP P2X7 receptors (P2X7Rs) exert pro-inflammatory effects. The aim of this study was to assess if there were differences in allelic and genotypic frequencies of a loss-of-function SNP of ADORA2A (rs2298383) and a gain-of-function single nucleotide polymorphism (SNP) of P2RX7 (rs208294) in the severity of SARS-CoV-2-associated infection. Fifty-five individuals were enrolled and categorized according to the severity of the infection. Endpoint genotyping was performed in blood cells to screen for both SNPs. The TT genotype (vs. CT + CC) and the T allele (vs. C allele) of P2RX7 SNP were found to be associated with more severe forms of COVID-19, whereas the association between ADORA2A SNP and the severity of infection was not significantly different. The T allele of P2RX7 SNP was more frequent in people with more than one comorbidity and with cardiovascular conditions and was associated with colorectal cancer. Our findings suggest a more prominent role of P2X7R rather than of A2AR polymorphisms in SARS-CoV-2 infection, although larger population-based studies should be performed to validate our conclusions.

## 1. Introduction

Coronavirus Disease-2019 (COVID-19) is caused by SARS-CoV-2, which triggers an infection of variable intensity resulting in five outcomes with increasing severity: asymptomatic, mild, moderate, severe and death [1]. COVID-19 starts with a pulmonary infection that can evolve into different complications, namely, acute respiratory distress syndrome (ARDS) but also cardiac or neurologic conditions, among others [2], which can either regress to normal or result in long-term physical or neuropsychiatric sequelae [3]. The severity of COVID-19 is related to the inflammatory response, whereby a controlled response allows recovery, whereas a deregulated hyperinflammatory state is associated with more severe presentations characterized by a cytokine storm [4], leading to ARDS and systemic complications [5].

The purinergic system plays an important role in the control of the immune-inflammatory system [6,7] and, accordingly, in the pathophysiology of various human diseases [8,9]. Purinergic receptors encompass two major families: P1 receptors (P1Rs) activated by adenosine and P2R activated by purine and pyrimidine nucleotides [8,9]. Both P1R and P2R are present in most immune cells and control inflammatory responses by contributing to the chemotaxis, activation, proliferation and differentiation of immune cells [6,7]. In particular, ATP P2X7 receptors (P2X7Rs) are a trigger and amplifier of inflammation [10], whereas adenosine A_2A_ receptors (A2ARs) are a major STOP signal of inflammation [11].

P2X7Rs are involved in different inflammatory pathways, particularly in the NLRP3 inflammasome, and control the intensity and the duration of inflammatory responses [6,10], contributing to exacerbated inflammatory responses in infectious environments, such as sepsis [12]. Concerning lung infections in particular, P2X7Rs play a role in aggressive forms of tuberculosis, in LPS (lipopolysaccharide)-induced lung inflammation and in the deleterious hyperinflammatory responses caused by *Influenza* virus infections [13,14,15,16], while being also responsible for decreasing surfactant production, worsening edema and acute lung injury [17]. Notably, the gene encoding P2X7R is highly polymorphic, and numerous SNPs (single nucleotide polymorphisms) have been identified and associated with human diseases, such as bipolar disorders, major depressive disorder, tuberculosis, *Chlamydia trachomatis* infection, among others [18]. The rs208294 SNP is a gain-of-function polymorphism of P2X7R that is linked to the pathophysiology of Alzheimer’s disease, systemic lupus erythematous complicated with pericarditis, as well as some infectious diseases like tuberculosis and HHV-6 infection (e.g., [19,20,21]).

A2AR are G protein-coupled receptors triggering anti-inflammatory effects [11] and reducing the severity of inflammation and reperfusion injury in various tissues in systemic inflammatory response syndromes like sepsis [22]. The immunosuppressive activity of A2AR is also involved in infection, as heralded by its protection against gut dysbiosis by *Candida albicans* [23] and in lung infection by *Escherichia coli*, *Klebsiella pneumoniae* and LPS-induced inflammation (e.g., [24,25,26]). ADORA2A, the gene encoding A2AR, has several polymorphisms that have been associated with anxiety, depression or cardiovascular disorders [27]. In particular, the rs2298383 SNP of ADORA2A is associated with higher production of pro-inflammatory cytokines and with aberrant immune activation [28].

This association of P2X7R and of A2AR with inflammation and infection has prompted multiple opinion papers hypothesizing that the antagonism of P2X7R [29,30,31,32] or activation of A2AR [32,33,34] may afford a benefit to manage COVID-19. However, apart from three reports describing that inhaled adenosine seems beneficial in patients with COVID-19-pneumonia [35], that an A2AR agonist regadenoson reduces inflammatory burden in 5 COVID-19 patients [36], that P2X7R controls the replication of the alpha SARS-CoV-2 variants [37] and that soluble P2X7R is increased in the plasma of COVID-19 patients [38], there is scarce experimental evidence linking P2X7R or A2AR to the severity of COVID-19.

To fill this gap of knowledge, we now investigated if the rs2298383 SNP of ADORA2A, associated with decreased A2AR activity [28], and the rs208294 SNP of P2RX7, associated with increased P2X7R activity [19,20,21], were linked to the severity of COVID-19.

## 2. Results

### 2.1. Demographic and Clinical Characteristics of the Subjects

This study included 55 subjects; among these, 4 were asymptomatic, 42 had mild disease, 3 had moderate disease and 6 had severe disease. Groups (asymptomatic + mild vs. moderate + severe) were similar in terms of sex, age, incidence of comorbidities and usual medications. As expected, there were differences regarding sequelae (*p* = 0.002) when comparing lower grades of severity with higher ones. The most common comorbidities included cardiovascular conditions (n = 29), particularly hypertension (n = 22) and dyslipidemia (n = 14), cancer (n = 29), particularly mammary cancer (n = 11) and colorectal cancer (n = 8), and neuropsychiatric conditions (n = 21), particularly depression (n = 13). The results of this section are summarized in Table 1, and the detailed list of comorbidities is presented in the Supplementary Materials (Table S1).

### 2.2. Impact of rs2298383 SNP of ADORA2A and of rs208294 SNP of P2RX7 in the Severity of SARS-CoV-2 Infection

All subjects were genotyped for the rs2298383 SNP of ADORA2A and for the rs208294 SNP of P2RX7 (Table 2). Genotype distributions did not deviate from the Hardy–Weinberg equilibrium (HWE) for the ADORA2A SNP (*p* = 1.000) and for the P2RX7 SNP (*p* = 0.477).

Genotypic and allelic frequencies of ADORA2A did not differ significantly between categories. Regarding P2X7R, there were statistically significant differences (*p* < 0.05) in the recessive model and in the allele frequencies. Using a binary logistic regression model, it was concluded that the T/T genotype of P2RX7 was six times more likely to be present in more severe patients (moderate + severe group) than C/C and C/T genotypes (*p* = 0.022, OR = 5.93, 95% CI 1.30–27.14). Also, the T allele was three times more likely to be present in more severe patients (moderate + severe group) than the C allele (*p* = 0.045, OR = 2.97, 95% CI 1.03–8.62).

### 2.3. Impact of the Combined Genotypes of Both SNPs in the Severity of SARS-CoV-2 Infection

To assess whether the combined ADORA2A SNP and P2RX7 SNP genotype influenced the severity of the infection, a new variable was created, taking in consideration all possible pairs. The results were not statistically significant (Table 3).

### 2.4. Impact of rs2298383 SNP of ADORA2A and of rs208294 SNP of P2RX7 in the Development of Comorbidities

After confirming that our cohort displayed statistically significant differences regarding age and the presence of comorbidities (*p* < 0.05), but not for the variable sex (*p* > 0.05), adjustments were only made for the variable age. Thus, age was considered a covariate in the logistic regression model.

In the multivariable analysis, the T allele (vs. C allele) of rs208294 SNP of P2RX7 (Table 4) was found to be more frequent in people with more than one comorbidity (*p* = 0.049, OR = 2.77, 95% CI 1.00–7.66). The remaining analyses did not reveal any association between the presence of at least one comorbidity and any of the genetic models (*p* > 0.05), when adjusting for age. Some results were initially significant but turned out to be non-significant in the multivariable analysis (Table 4).

As for the combination of the genotypes of both SNPs, the results were also non-significant in the multivariable analysis (Table 5).

An analysis of the possible impact of these SNPs in the development of specific conditions was also performed, considering only the most prevalent comorbidities in the studied population. First, it was confirmed that there were significant differences when comparing age with cardiovascular conditions, hypertension and dyslipidemia (*p* < 0.05). There were also significant differences when comparing sex with mammary cancer (*p* < 0.05). Adjustments for age and sex were performed attending to these results, considering these variables as covariates in logistic regression models.

As for the rs2298383 SNP of ADORA2A, differences were observed solely for hypertension in the general and dominant models. However, when considering age as a covariate in the multivariable analysis, the result was non-significant, even though it was marginal (*p* = 0.055, OR = 5.355, 95% CI = 0.962–29.816) for the C/T genotype. Analyses of the recessive model and allele frequency of rs2298383 SNP of ADORA2A did not reveal any statistical difference.

As for rs208294 SNP of P2RX7, differences were observed regarding cardiovascular conditions and colorectal cancer. For the general and dominant models, there were statistically significant results regarding colorectal cancer (*p* = 0.046 and *p* = 0.040). The odds ratios are not shown, because the logistic regression analysis was non-significant. Furthermore, the T allele was more frequent in people presenting cardiovascular conditions, even when considering age as a covariate in a logistic regression model. Subjects presenting the T allele were three times more likely to have a cardiovascular condition (*p* = 0.043, OR = 3.460, 95% CI 1.04–11.55).

As for the combined genotypes of the two SNPs, the bivariate analysis showed statistical significance for hypertension (*p* = 0.006). Nevertheless, the result was non-significant, after adjustment for age in the multivariable analysis. The results of this section are summarized in Table 6.

## 3. Discussion

The present study provides the first results relating the severity and the incidence of co-morbidities of COVID-19 with two polymorphisms of two purinergic receptors, P2X7R and A2AR, involved in the control of inflammation and tissue damage [6,7]. Our findings highlighted the particular association of the rs208294 SNP of P2RX7, corresponding to a gain of function of P2X7R [19,20,21], with more severe forms of COVID-19, being more frequently found in patients with two or more comorbidities and particularly associated with co-morbidities such as cardiovascular conditions and colorectal cancer. Nevertheless, this study should be considered as a proof-of-concept of the role of the A2AR and P2X7R polymorphisms in the probability of developing severe disease due to SARS-CoV-2 infection, since the sample size is limited (n = 55), which may affect the power to identify statistically significant associations in comparison with larger data sets. In spite of its limited size, the cohort used in this study that included only patients from the central region of Portugal was considered homogenous since the different groups did not display significant differences in the distribution of age, sex, presence of comorbidities, medication and genetic background. Testing for Hardy–Weinberg equilibrium (HWE) was performed for the results of both SNPs, and deviations were not observed, indicating that the sampling was obtained from a general population without effects of natural selection, migration, mutation or random drift [39].

Although the role of A2AR in the severity of SARS-CoV-2 infection has been hypothesized [32,33,34] and preliminary clinical data indicate that the infusion of either adenosine or of a selective A2AR agonist may dampen inflammation in COVID-19 patients [35,36], our study did not reveal any association between SARS-CoV-2 infection and the rs2298383 SNP of A2AR, a loss-of-function polymorphism associated with bolstered immune response [28]. We have previously shown in the context of brain diseases that the altered role of A2AR depends both on the up-regulation of A2AR as well as an increased formation of extracellular adenosine [40,41,42,43]. Thus, the contrasting evidence that A2AR properties seems unrelated to the severity of COVID-19 whereas exogenously added agonists provide a clinical benefit [35,36] is suggestive that the major modification in the adenosine system associated with COVID-19 might be the availability of extracellular adenosine. Future studies assessing the levels of extracellular adenosine as well as the function of A2AR during infection, or of other SNPs of A2AR [27], are needed to better understand a possible role of the adenosine A2AR modulation system in SARS-CoV-2 pathophysiology.

In contrast, our results revealed a significant association of the rs208294 SNP of P2RX7 with the severity of COVID-19. Thus, the TT genotype was associated with an increased risk of more severe disease (vs. TC + CC), and accordingly, the T allele showed the same effect (vs. the C allele). This leads to the contention that this SNP may be a potential biomarker of developing a more severe disease, although further studies are needed to validate this conclusion. The 489C>T SNP of P2RX7 corresponds to a gain-of-function polymorphism, being potentially associated with enhanced and inappropriate pro-inflammatory environment during SARS-CoV-2 infection, which is a determinant of COVID-19 severity [4]. Our studies may be in accordance with this scenario by pointing to the association between the TT genotype and moderate-severe disease.

The comorbidities registered in our cohort were similar to the sequelae-related differences generally reported in epidemiological studies about COVID-19 [5]. The multivariable analysis indicated that the SNP of ADORA2A was not a determinant for the development of comorbidities, whereas the T allele of P2RX7 SNP was more frequently found in patients with two or more comorbidities. This finding is easily understandable based on the previous reports that this T allele of P2RX7 influences the severity of infection, and gain-of-function SNPs of P2RX7 are related to severity of multiple inflammation-associated diseases [19,20,21]. Hence, P2X7R may be both a direct (during infection) and an indirect factor (by increasing the number of comorbidities) affecting SARS-CoV-2 severity, which is in line with the reported increased plasma levels of P2X7R associated with the severity of COVID-19 [38].

Amongst the most prevalent comorbidities, our general and dominant models showed that the gain-of-function T allele of P2RX7 SNP was more frequent in COVID-19 patients with cardiovascular conditions, which mirrors the previously reported association between a P2RX7 loss-of-function SNP and a reduced risk of cardiovascular events [44]. The analyses also indicated an association between the distribution of the rs208294 SNP of P2RX7 and the incidence of colorectal cancer in COVID-19 patients, as observed in the general population [45]. This aligns with the increasing awareness of the role of P2X7R in carcinogenesis and in the control of antitumor immune response (reviewed in [46]), translated into a booming number of patents and clinical trials with drugs targeting P2X7R in cancer [47]. Moreover, P2X7R polymorphisms seem to emerge as ancillary biomarkers of the risk and prognosis of cancers, as heralded by the observations that the C allele of the rs208294 SNP of P2RX7 has been associated with better prognosis in metastatic colorectal cancer [48,49], while the C/T genotype has been identified as a protective factor in other cancer situations, such as acute leukemia [48] or prostate cancer [50].

The database analysis of our data also provided an interesting observation with respect to the distribution of the studied SNPs of A2AR with age. Thus, it was noted that the CC genotype of ADORA2A SNP was under-represented in older individuals, in comparison with the CT and TT genotypes (*p* < 0.05). Thus, there is a linkage disequilibrium of A2AR polymorphisms on aging, which is particularly relevant in view of the established association of caffeine (which affords protection by blocking A2AR [51]) with the quality of life with aging [52,53,54] and the recently described ability of A2AR to control senescence [55]. Future studies will be required to confirm this association as well as the proposed association of P2RX7 polymorphisms with aging in Caucasian populations [56].

In conclusion, the present study provides the first proof-of-concept supporting an association between genetic variants of purinergic receptors, in particular, the rs208294 SNP of P2RX7, and the severity of SARS-CoV-2 infection. However, larger population-based studies and well-powered studies should be performed to validate the preliminary conclusions obtained with this exploratory work.

## 4. Materials and Methods

### 4.1. Study Design and Subjects

This analytical cohort study included individuals recovered from SARS-CoV-2 infection aged 18 and above, and the selection was based on the clinicians’ interview of the participants. The recruitment of volunteers was carried out at Instituto Português de Oncologia de Coimbra Francisco Gentil, EPE (IPO), at IPSS—Casa do Pai and at Escola Superior de Saúde Dr. Lopes Dias. It was held from March 2021 to November 2021. These volunteer participants had to be asymptomatic for at least 3 days before the collection of the sample, and previous SARS-CoV-2 infection was considered only if with a positive RT-PCR diagnosis. The blood sample was obtained when patients attended a routine medical appointment or treatment, as well as from employees of IPO. The only exclusion criterium was not having documented SARS-CoV-2 infection. Fifty-five volunteers were enrolled. Demographic and clinical data were obtained from the interview and from the medical file. Demographic data included sex and age; clinical parameters included the symptoms during the disease and the sequelae of the infection, as well as comorbidities and usual medications. This study included individuals with a distinct clinical spectrum of COVID-19, i.e., of severity of SARS-CoV-2 infection, categorized in four groups, according to previous studies [1]. The criteria defining the groups were based on a study by Maier et al. [57] and are presented in the Supplementary Materials (Table S2). An adaptation of the categorization was made, excluding the self-reported severity of symptoms and redundant concepts. Regarding sequelae, patients were categorized in three groups: recovered, with sequelae or deceased. A database was constructed gathering the collected information, and a biobank was built with the whole venous blood samples.

### 4.2. Isolation and Extraction of Genomic DNA

Genomic DNA was isolated from the collected whole venous blood samples and stored frozen at −80 °C, in EDTA-treated tubes. For the extraction of DNA, the NucleoSpin Blood kit (MACHEREY-NAGEL, Düren, Germany) was used according to manufacturer’s instructions. The DNA concentration and purity were measured using a NanoDrop 2000 (Thermo Fisher Scientific, Waltham, MA, USA), followed by storage at −20 °C, until genotyping.

### 4.3. SNPs Selection

The rs2298383 SNP of ADORA2A [28] and the rs208294 SNP of P2RX7 [19,20,21] were selected according to previous reports of association of the chosen SNPs with inflammatory disorders and infectious diseases, as well as the predicted gene product activity in SARS-CoV-2 infection.

### 4.4. Genotyping

The screening of the selected SNPs was performed by endpoint genotyping with a LightCycler^®^480 Real-Time PCR System (Roche, Basel, Switzerland), version 1.50, using two validated probes (Thermo Fisher Scientific, Waltham, MA, USA) for each SNP, according to the manufacturer’s protocol. Regarding gene amplification, two validated primers (Thermo Fisher Scientific, Waltham, MA, USA) were used for each SNP. Individually, 10.0 µL of reaction contained 2.5 µL of genomic DNA in a concentration of approximately 3.5 ng/µL, 0.5 µL of TaqMan^®^ SNP Genotyping Assay (Thermo Fisher Scientific, Waltham, MA, USA), 5.0 µL of TaqPath™ ProAmp™ Master Mix (Thermo Fisher Scientific, Waltham, MA, USA) and 2.0 µL of nuclease-free water. Positive and negative controls were used in all the experiments, and each sample was analyzed in duplicate for quality control. The same thermal program was applied for both SNPs in all the experiments. The amplified sequences are presented in Table 7. The results were interpreted according to the endpoint fluorescence. The genotype determination was blind in what regards the demographic and clinical data.

### 4.5. Statistical Analysis

Data are presented as mean ± standard deviation and as absolute and relative frequencies for continuous and categorical variables, respectively. Fisher’s exact test or Fisher–Freeman–Halton exact test was used, as appropriate, to assess differences in the distribution of sex, sequels, comorbidities and usual medications in the categories of the severity of infection. Mann–Whitney U test was used for assessing the distribution of age because its distribution was not normal as revealed by the Shapiro–Wilk test. A Chi-square test was used for testing the compliance of the genotype’s distributions to the Hardy–Weinberg equilibrium (HWE). Fisher’s exact test, Fisher–Freeman–Halton exact test or Chi-square test were used to determine, as appropriate, if there was an association between allelic and genotypic frequencies and the severity groups. To evaluate the impact of the considered SNPs in the development of comorbidities, Fisher’s exact test, Fisher–Freeman–Halton exact test or Chi-square test was used accordingly for nominal/categorical variables. Kruskal–Wallis or Mann–Whitney U test was used accordingly for continuous variables. Logistic regression modeling was used to understand the relationship between the variables and to adjust the results to possible covariates. This way, in multivariable analyses, covariates were included in logistic regression models to control its effects on the dependent outcome variable, eliminating alternative explanations. Chi-square test was used when Cochran’s rules were fulfilled. A *p*-value lower than 0.05 was considered as statistically significant.

## Figures and Tables

**Table 1:** Demographic and clinical characteristics of the study populations.

		Severity				
		Asymptomatic + Mild		Moderate + Severe		
		Frequency ^1^	Mean ^2^	Frequency ^1^	Mean ^2^	*p*-Value ^3^
Sex	**Male**	28.3% (13)		66.7% (6)		0.051
	**Female**	71.7% (33)		33.3% (3)		
Age			65.0 (±20.4)		65.7 (±18.6)	0.964
Sequelae	**Recovered**	84.8% (39)		0% (0)		**0.002**
	**Physical and/or Psychological**	15.2% (7)		22.2% (2)		
	**Death**	0% (0)		11.1% (1)		
Comorbidities	**Absence**	10.9% (5)		11.1% (1)		1.000
	**Presence**	89.1% (41)		88.9% (8)		
Usual Medications	**Absence**	26.7% (12)		37.5% (3)		0.673
	**Presence**	73.3% (33)		62.5% (5)		
Total		83.6% (46)		16.4% (9)		

^1^ For categorical variables, values are presented as % (n). ^2^ For continuous variables, values are presented as mean (±standard deviation). ^3^ Bold cells indicate statistically significant differences. Fisher’s exact test and Fisher–Freeman–Halton exact test were used for nominal/categorical variables. Mann–Whitney U test was used for age vs. severity.

**Table 2:** Genotypic and allelic frequencies of ADORA2A rs2298383 SNP and of P2RX7 rs208294 SNP in the different categories of severity of infection, according to different genetic models [values are presented as % (n)].

ADORA2A rs2298383 SNP				
		Asymptomatic + Mild ^1^	Moderate + Severe ^1^	*p*-Value ^2^
General model	**C/C**	17.4% (8)	22.2% (2)	0.552
	**C/T**	52.2% (24)	33.3% (3)	
	**T/T**	30.4% (14)	44.4% (4)	
Dominant model	**C/T; T/T**	82.6% (38)	77.8% (7)	0.661
	**C/C**	17.4% (8)	22.2% (2)	
Recessive model	**T/T**	30.4% (14)	44.4% (4)	0.454
	**C/T; C/C**	69.6% (32)	55.6% (5)	
Allele	**T allele frequency**	56.5%	61.1%	0.719
	**C allele frequency**	43.5%	38.9%	
**P2RX7 rs208294 SNP**				
		**Asymptomatic + Mild ^1^**	**Moderate + Severe ^1^**	***p*-Value ^2^**
General model	**C/C**	37.0% (17)	22.2% (2)	0.070
	**C/T**	45.7% (21)	22.2% (2)	
	**T/T**	17.4% (8)	55.6% (5)	
Dominant model	**C/T; T/T**	63.0% (29)	77.8% (7)	0.473
	**C/C**	37.0% (17)	22.2% (2)	
Recessive model	**T/T**	17.4% (8)	55.6% (5)	0.026 *
	**C/T; C/C**	82.6% (38)	44.4% (4)	
Allele	**T allele frequency**	40.2%	66.7%	0.039 **
	**C allele frequency**	59.8%	33.3%	

^1^ Values are presented as % (n) for the general, dominant and recessive models, and as % for allele frequency. ^2^ Bold cells indicate statistically significant differences. Fisher’s exact test, Fisher–Freeman–Halton exact test and Chi-square test were used accordingly. * Odds ratio (OR) = 5.93 with 95% CI 1.30–27.14, calculated with binary logistic regression (*p* = 0.022). ** Odds ratio (OR) = 2.97 with 95% CI 1.03–8.62, calculated with binary logistic regression (*p* = 0.045).

**Table 3:** Distribution of the combined genotypes of the SNPs in the different categories of severity of infection [values are presented as % (n)].

Combined Genotypes of Both SNPs	Asymptomatic + Mild	Moderate + Severe	*p*-Value ^1^
P2X7R—T/T A2AR—T/T	2.2% (1)	11.1% (1)	0.166
P2X7R—T/T A2AR—T/C	6.5% (3)	22.2% (2)	
P2X7R—T/T A2AR—C/C	8.7% (4)	22.2% (2)	
P2X7R—T/C A2AR—T/T	13.0% (6)	22.2% (2)	
P2X7R—T/C A2AR—T/C	30.4% (14)	0% (0)	
P2X7R—T/C A2AR—C/C	2.2% (1)	0% (0)	
P2X7R—C/C A2AR—T/T	15.2% (7)	11.1% (1)	
P2X7R—C/C A2AR—T/C	15.2% (7)	11.1% (1)	
P2X7R—C/C A2AR—C/C	6.5% (3)	0% (0)	

^1^ Using Fisher–Freeman–Halton exact test.

**Table 4:** Results (*p*-value) of the analysis of genotypic and allelic frequencies of either ADORA2A rs2298383 SNP or P2RX7 rs208294 SNP regarding the presence of comorbidities, according to different genetic models.

ADORA2A rs2298383 SNP					
	General Model ^1^	Dominant Model ^1^	Recessive Model ^1^	Allele ^1^	Multivariable Analysis ^2^
	TT vs. CT vs CC	TT; CT vs. CC	TT vs. CT; CC	T vs. C	
Presence of comorbidities	0.067	0.066	1.000	0.327	NS
Number of comorbidities	**0.018**	**0.020**	0.466	0.437	NS
0, 1 or >1 comorbidity	0.113	0.072	0.774	0.507	NS
**P2RX7 rs208294 SNP**					
	**General Model ^1^**	**Dominant Model ^1^**	**Recessive Model ^1^**	**Allele ^1^**	**Multivariable** **Analysis ^2^**
	**TT vs. CT** **vs CC**	**TT; CT vs. CC**	**TT vs. CT; CC**	**T vs. C**	
Presence of comorbidities	0.174	0.167	0.317	0.181	NS
Number of comorbidities	0.153	0.053	0.417	0.070	NS
0, 1 or >1 comorbidity	0.138	**0.043**	0.438	**0.047**	Sig. *

Abbreviations: Sig, significant result. NS, non-significant result. ^1^ Bold cells indicate statistically significant differences. For the variables “Presence of comorbidities” and “0, 1 or >1 comorbidity”, Fisher’s exact test, Fisher–Freeman–Halton exact test or Chi-square test was used. For the variable “Number of comorbidities”, Kruskal–Wallis or Mann–Whitney U test was used accordingly. ^2^ Bold cells indicate statistically significant differences. For the multivariable analysis, the results were adjusted for age (considered as a covariate) in a logistic regression model. * Odds ratio (OR) = 2.77 with 95% CI 1.00–7.66, calculated with binary logistic regression and considering age as a covariate (*p* = 0.049), for allele frequency of P2RX7 SNP vs. having 0, 1 or more than 1 comorbidity.

**Table 5:** Results (*p*-value) of the analysis of the combined genotypes of the SNPs regarding the presence of comorbidities.

	*p*-Value ^1^	Multivariable Analysis ^2^
Presence of comorbidities	**0.015**	NS
Number of comorbidities	**0.021**	NS
0, 1 or >1 comorbidity	**0.030**	NS

^1^ Bold cells indicate statistically significant differences, when using Fisher–Freeman–Halton exact test for the variables “Presence of comorbidities” or “0, 1 or >1 comorbidity” and Kruskal–Wallis test for the variable “Number of comorbidities”. ^2^ For the multivariable analysis, the results were adjusted for age (considered as a covariate) in a logistic regression model. NS, non-significant result.

**Table 6:** Results (*p*-value) of the analysis of genotypic and allelic frequencies of rs2298383 SNP of ADORA2A and of rs208294 SNP of P2RX7 regarding the presence of specific conditions, according to different genetic models.

	ADORA2A rs2298383 SNP				P2RX7 rs208294 SNP					
	Gen. Model ^1^	Dom. Model ^1^	Rec. Model ^1^	Allele ^1^	Gen. Model ^1^	Dom. Model ^1^	Rec. Model ^1^	Allele ^1^	Combined Genotypes ^1^	Multivariable Analysis ^2^
	T/T vs. C/T vs. C/C	T/T; C/T vs. C/C	T/T vs. C/T; C/C	T vs. C	T/T vs. C/T vs. C/C	T/T; C/T vs. C/C	T/T vs. C/T; C/C	T vs. C		
Cardiovascular conditions	0.097	0.164	0.391	0.763	0.181	0.086	0.215	**0.047**	0.102	**Sig. ^3^**
Hypertension	**0.011**	**0.039**	0.197	0.753	0.643	0.354	0.604	0.347	**0.006**	**NS ^4^**
Dyslipidemia	0.056	0.423	0.110	0.647	0.299	0.334	0.218	0.120	0.221	-
Cancer	0.328	0.490	0.391	0.933	0.497	0.577	0.238	0.276	0.421	-
Mammary cancer	0.907	1.000	1.000	0.772	0.524	1.000	0.266	0.388	0.934	-
Colorectal cancer	0.882	1.000	1.000	0.929	**0.046**	**0.040**	1.000	0.118	0.202	-
Neuropsychiatric conditions	0.131	0.287	0.268	0.983	0.184	0.082	0.498	0.090	0.235	-
Depression	0.119	0.095	1.000	0.339	0.366	0.734	0.156	0.244	0.501	-

Abbreviations: Sig, significant result. NS, non-significant result. Gen, General. Dom, Dominant. Rec, Recessive. ^1^ Bold cells indicate statistically significant differences. Fisher’s exact test, Fisher–Freeman–Halton exact test and Chi-square test were used accordingly. ^2^ Bold cells indicate statistically significant differences. For the multivariable analysis, the results were adjusted for age and/or sex (considered as covariates) in a logistic regression model. These results are only present when the bivariate analysis is statistically significant. As for colorectal cancer, age and sex were not considered as covariates. ^3^ Odds ratio (OR) = 3.46 with 95% CI 1.04–11.55, calculated with binary logistic regression and considering age as a covariate (*p* = 0.043), for allele frequency of P2RX7 SNP vs. Cardiovascular conditions. ^4^ The results were non-significant, after adjustment for age with logistic regression.

**Table 7:** Studied SNPs and representation of the amplified sequences.

Gene	SNP	5′-3′ Sequence
ADORA2A	rs2298383	TGC TTT GAC CCC TAT AGG AAT TCA GAC CGG AAG GTG TGT AGT **G[C/T]A** TGA AGG GAA CCA GAA GAC CTG TGA AGT CTC TGC CTG GTG
P2RX7	rs208294	CCA GAT CCT GGC CCC GCC CCC TCC **C[C/T]G** GGG CCT CTG ACC TTC CTG TCA CTC

## Data Availability

Data are contained within the article or Supplementary Materials.

## Supplementary Materials

The following supporting information can be downloaded at: https://www.mdpi.com/article/10.3390/ijms25116135/s1.
