# Supplementary material for: Genetic Polymorphisms of P2RX7 but Not of ADORA2A Are Associated with the Severity of SARS-CoV-2 Infection
Source: Int J Mol Sci. 2024 Jun 2;25(11):6135. doi: 10.3390/ijms25116135 (PMC11173306; doi:10.3390/ijms25116135)
Supplement: Supplementary file 1 [file ijms-25-06135-s001.zip › ijms-3007878-supplementary.pdf]

Supporting Information

Table S1. Review of the comorbidities present in the study population.

**Comorbidities[1]**	
**Cardiovascular conditions**	**52.7% (29)**
Hypertension	40.0% (22)
Dyslipidemia	25.5% (14)
Cardiac arrythmias	9.1% (5)
Cardiac ischemic disease	7.3% (4)
Diabetes	7.3% (4)
Valvular heart disease	5.5% (3)
Obesity	5.5% (3)
Heart failure	3.6% (2)
Stroke	3.6% (2)
Chronic venous disease	1.8% (1)
**Oncologic conditions**	**52.7% (29)**
Mammary cancer	20.0% (11)
Colorectal cancer	14.6% (8)
Melanoma	3.6% (2)
Carcinoma of unknown primary origin	3.6% (2)
Lymphoma	1.8% (1)
Gastric neoplasia	1.8% (1)
Pancreatic neoplasia	1.8% (1)
Astrocytoma	1.8% (1)

Lung neoplasia	1.8% (1)
Multiple myeloma	1.8% (1)
Prostate cancer	1.8% (1)
**Neuropsychiatric conditions**	**38.2% (21)**
Depression	23.6% (13)
Dementia	9.09% (5)
Epilepsy	5.45% (3)
Schizophrenia	1.8% (1)
Anxiety disorder	1.8% (1)
**Miscellaneous**	
Hypothyroidism	7.3% (4)
Degenerative joint disease	5.5% (3)
Anemia	5.5% (3)
Chronic pulmonary disorders	5.5% (3)
Cataract	5.5% (3)
Glaucoma	3.6% (2)
Benign prostatic hyperplasia	3.6% (2)
Vertiginous syndrome	3.6% (2)
Hypoacusis	3.6% (2)
Chronic kidney disease	1.8% (1)
Lactose intolerance	1.8% (1)
Alopecia	1.8% (1)

[1] Values are expressed as %(n).

Table S2. Degrees of severity of SARS-CoV-2 infection and criteria applied to define each group.

**Asymptomatic**	**Mild**	**Moderate**	**Severe**
Absence of symptoms related to SARS-CoV-2 infection	Anosmia/Ageusia / 2 or more COVID-19-manifestations (below)	More severe symptoms like:<br>- Dyspnea<br>- Tachypnea<br>- Chest tightness<br>- Chest pain	Hospital admission
Presence of symptoms not related to SARS-CoV-2 infection	Fever<br>Chills/shaking<br>Headache<br>Rhinorrhea<br>Nasal congestion<br>Cough<br>Pharyngitis or itchy throat<br>Arthralgia<br>Myalgia<br>Anorexia<br>Abdominal pain<br>Diarrhea<br>Vomit<br>Rash<br>Asthenia<br>Bedfast<br>Conjunctivitis<br>Insomnia<br>Syncope<br>Dizziness	Outpatient hospital visit	
